# Publisher Correction: Ca^2+^-regulated Ca^2+^ channels with an RCK gating ring control plant symbiotic associations

**DOI:** 10.1038/s41467-019-12590-y

**Published:** 2019-10-07

**Authors:** Sunghoon Kim, Weizhong Zeng, Shane Bernard, Jun Liao, Muthusubramanian Venkateshwaran, Jean-Michel Ane, Youxing Jiang

**Affiliations:** 1 Howard Hughes Medical Institute and Department of Physiology, University of Texas Southwestern Medical Center, Dallas, TX USA; 2 Department of Biophysics, University of Texas Southwestern Medical Center, Dallas, TX USA; 3 Department of Bacteriology, University of Wisconsin-Madison, Madison, WI USA; 4 School of Life Science and Technology, ShanghaiTech University, 201210 Shanghai, China; 5 School of Agriculture, University of Wisconsin-Platteville, Platteville, WI USA

**Keywords:** Ion transport, Rhizobial symbiosis, X-ray crystallography, Calcium channels

Correction to: *Nature Communications* 10.1038/s41467-019-11698-5, published online 16 August 2019.

The originally published version of this Article contained errors in Fig. 7c that were inadvertently introduced during typesetting. The label of third panel on the top row was inadvertently labelled DMI1 2TM rather than DMI1 4TM. Also, the second panel on the bottom row was inadvertently labelled D470A rather than D472A. These errors have now been corrected in both the PDF and HTML versions of the Article.

