# Supplementary material for: Ca2+-regulated Ca2+ channels with an RCK gating ring control plant symbiotic associations
Source: Nat Commun. 2019 Aug 16;10:3703. doi: 10.1038/s41467-019-11698-5 (PMC6697748; doi:10.1038/s41467-019-11698-5)
Supplement: Supplementary file 1 — Supplementary Information [file 41467_2019_11698_MOESM1_ESM.pdf]

Supplementary Information for:

Ca^{2+}-regulated Ca^{2+} channels with an RCK gating ring control plant symbiotic associations

Kim, S. et al.

Supplementary Table 1 Crystallographic Statistics

Data Set	Native (Sodium)	SAD (Se-Met)	Potassium	Apo state
**Data Collection**				
Space Group	*I4*	*I4*	*I4*	*P 2₁*
Cell Dimension				
a, b, c (Å)	125.2, 125.2, 81.5	125.1, 125.1, 82.0	125.6, 125.6, 82.8	100.4, 116.0, 113.0
α, β, γ (˚)	90.0, 90.0, 90.0	90.0, 90.0, 90.0	90.0, 90.0, 90.0	90.0, 113.9, 90.0
Wavelength (Å)	0.94936	0.97927	0.99996	1.03320
Resolution (Å)	50.00 – 1.60 (1.63 – 1.60)[a]	50.00 – 2.10 (2.14 – 2.10)	50.00 — 1.85 (1.88 – 1.85)	50.0 – 3.30 (3.36 – 3.30)
R_{merge}	0.075 (1.201)	0.129 (0.830)	0.063 (1.670)	0.083 (0.550)
I/σ	34.54 (1.98)	29.11 (3.91)	8.60 (1.00)	22.26 (2.92)
Completeness (%)	98.8 (90.2)	100.0 (100.0)	99.9 (100.0)	99.7 (98.6)
Redundancy	4.9 (3.8)	10.1 (10.2)	7.4 (7.2)	3.8 (3.8)
**Refinement**				
Resolution (Å)	37.16 - 1.60 (1.66 - 1.60)		37.52 – 1.85 (1.91-1.85)	41.46 – 3.30 (3.42 – 3.30)
No. of Unique Reflection	81466 (7449)		55044 (5355)	35798 (3514)
R_{work} / R_{free}	0.1659/0.1882		0.1734/0.2096	0.2290/0.2838
No. of atoms				
Protein	3984		3970	15882
Ligand	6		6	0
Water	534		293	0
B factors				
Protein	24.25		48.93	113.15
Ligand	17.06		37.59	-
Water	35.92		51.92	-
R. m. s deviations				
Bond Lengths (Å)	0.011		0.020	0.006
Bond Angles (˚)	1.38		1.93	1.09
Ramachandran Plot				
Preferred (%)	99.60		99.40	97.16
Allowed (%)	0.60		0.60	2.59
Outliers (%)	0.00		0.00	0.25
Clash score	1.37		2.50	11.31

[a] The values in the parentheses are for the highest resolution shell

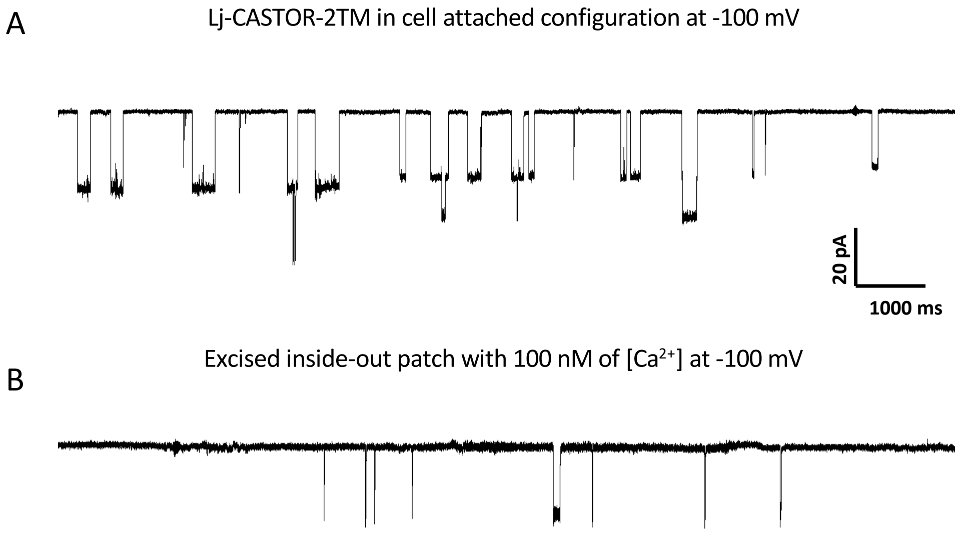

Supplementary Figure 1. LjCASTOR-2TM exhibits much higher single channel activity in the cell-attached patch with low endogenous [Ca^{2+}] **(A)** than that measured in the excised inside-out patch with 100 nM of [Ca^{2+}] in the bath solution **(B)**.

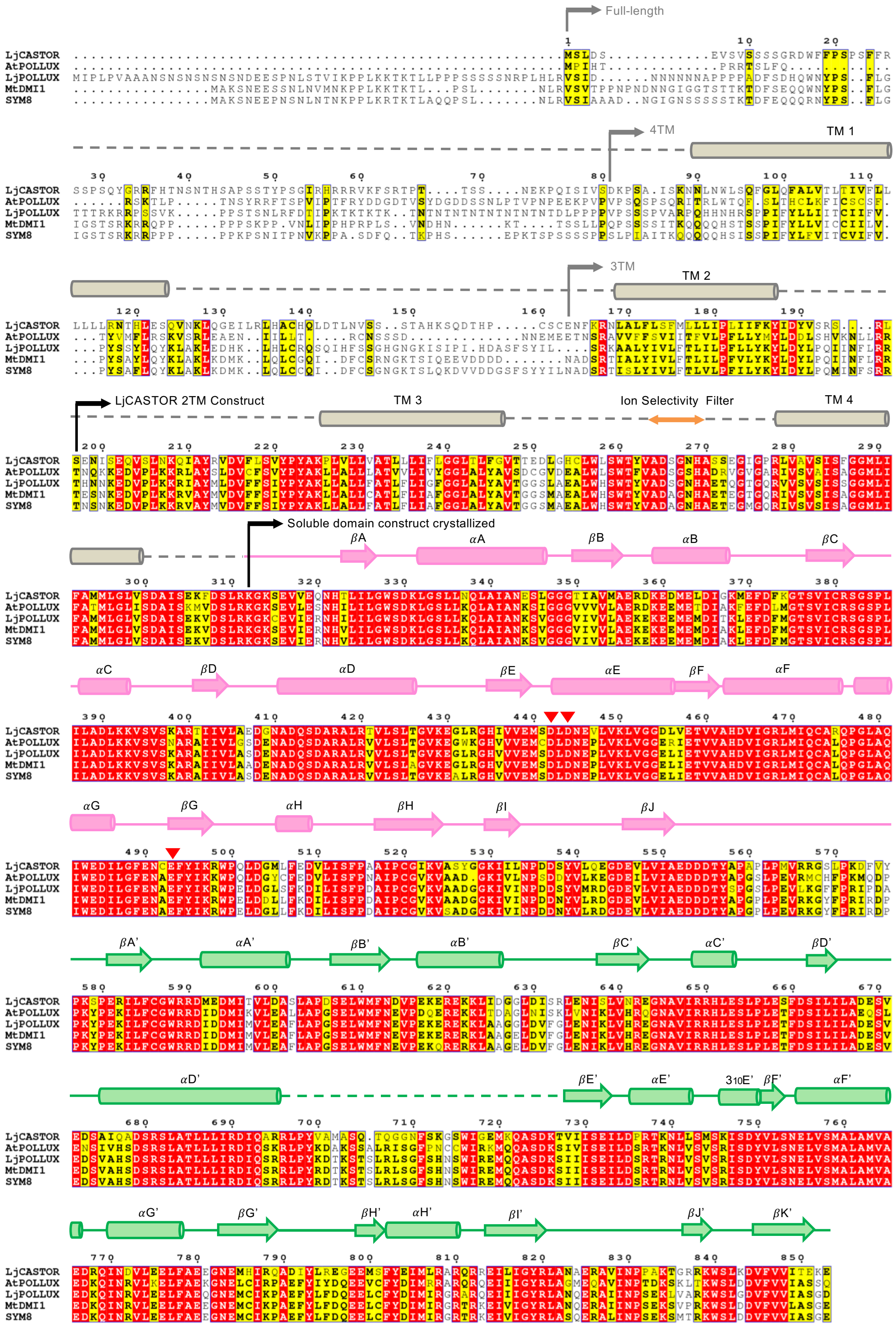

Supplementary Figure 2. Sequence alignment and secondary structure of LjCASTOR and its homologs. Amino acid sequence of LjCASTOR (UniProtKB entry code, Q5H8A6) is aligned with *Arabidopsis thaliana* POLLUX (AtPOLLUX, Q9LTX4), *Lotus japonicus* POLLUX (LjPOLLUX, Q5H8A5), *Medicago truncatula* DMI1 (MtDMI1, Q6RHR6), and *Pisum sativum* SYM8 (Q4VY51). Secondary structure assignment for the soluble domain is based on the crystal structure of LjCASTOR RCK domain. Red inverted triangles mark the key calcium-coordinating residues used for the mutation studies. The LjCASTOR 2TM construct used for the electrophysiological studies and the intracellular domain construct which has been crystallized are highlighted with black arrows. The constructs with the serial N-terminal truncations which are not functional in patch-clamp measurement are marked with gray arrows (Full-length, 4TM, and 3TM).

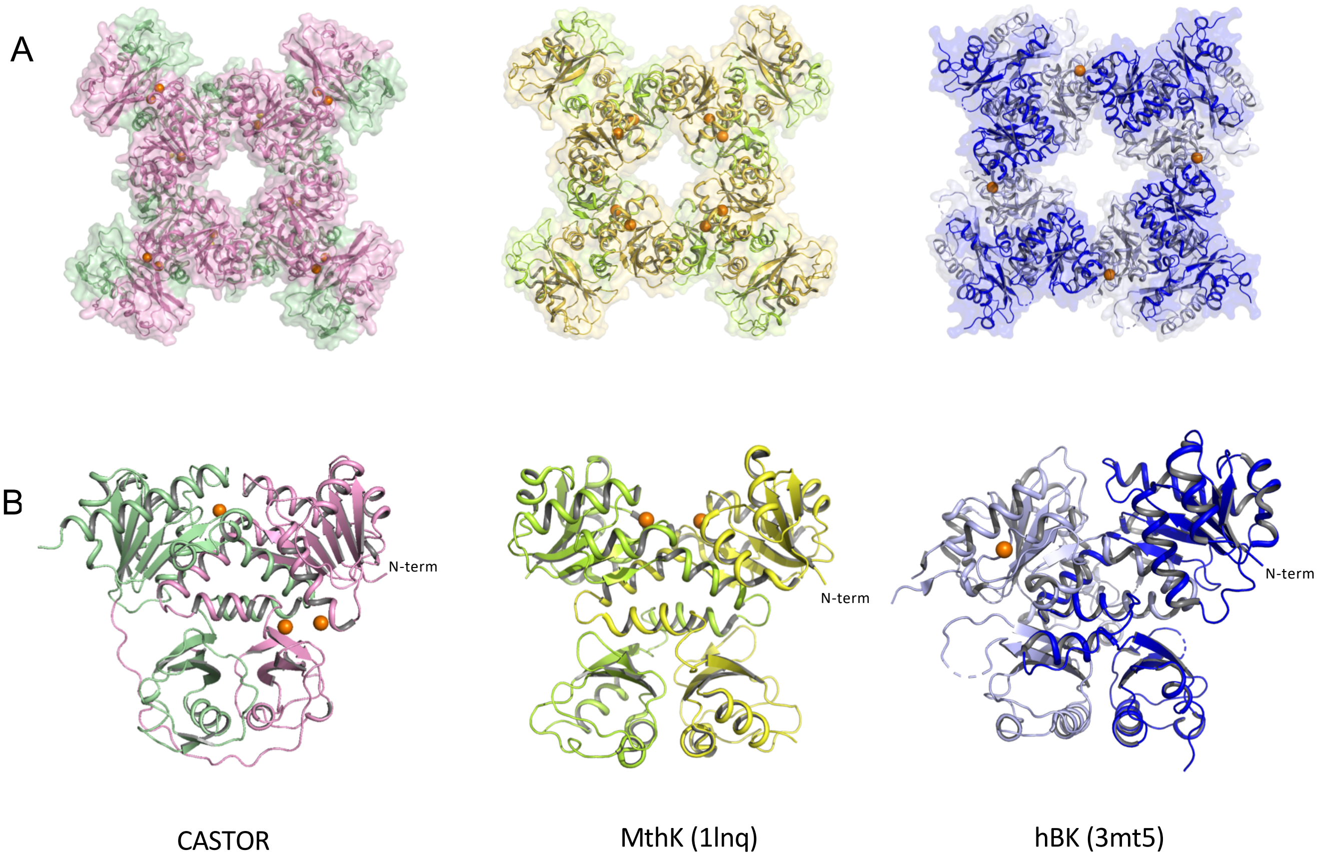

Supplementary Figure 3. Comparison of the Ca^{2+}-bound LjCASTOR gating ring structure with two representative RCK gating ring structures from prokaryotic MthK and human BK channels. The top view of the gating ring **(A)** and a single subunit structure **(B)** of the LjCASTOR (left, the same color scheme as in Figure 2A), MthK (middle, RCK1 in yellow and RCK2 in lime colors; PDB code, 1lnq) and human BK channel (right, RCK1 in blue and RCK2 in lightblue; PDB code, 3mt5) are placed for comparison. The bound calcium ions were displayed as orange spheres and all other ligands were ignored for visual clarity.

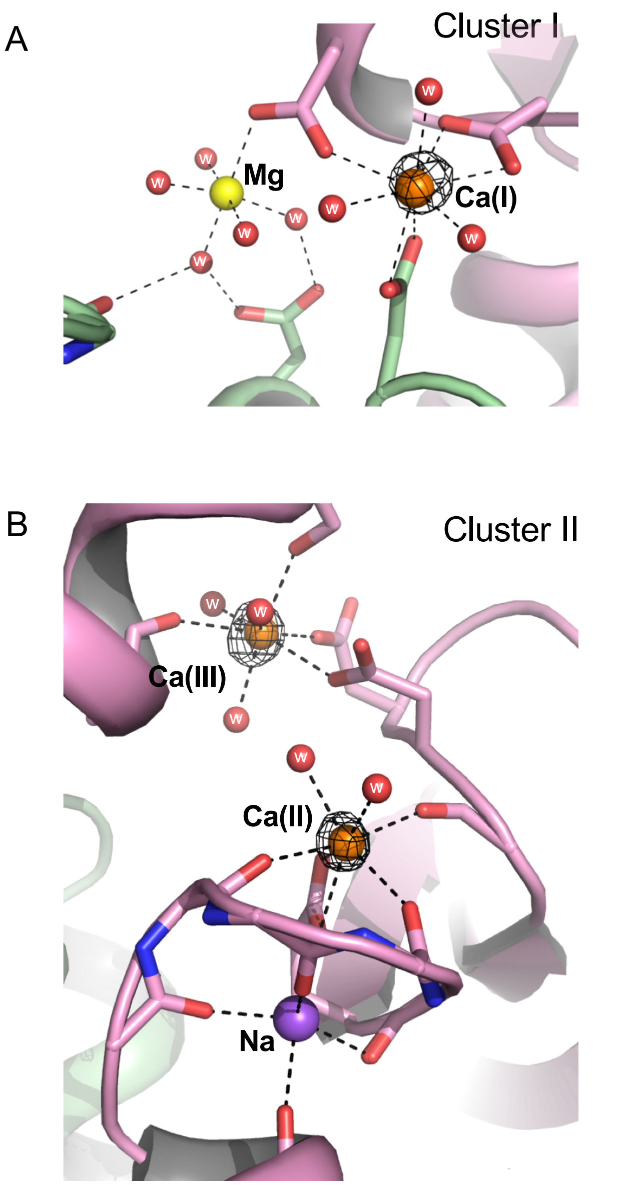

Anomalous difference map contoured at 5.0 σ

Supplementary Figure 4. Anomalous difference Fourier maps (grey mesh contoured at 5.0 σ) at the cluster I **(A)** and cluster II **(B)** ion binding sites. Anomalous scattering signals are observed only at the Ca^{2+}-binding sites (Ca(I), Ca(II), and Ca(III)) but not at Na and Mg sites.

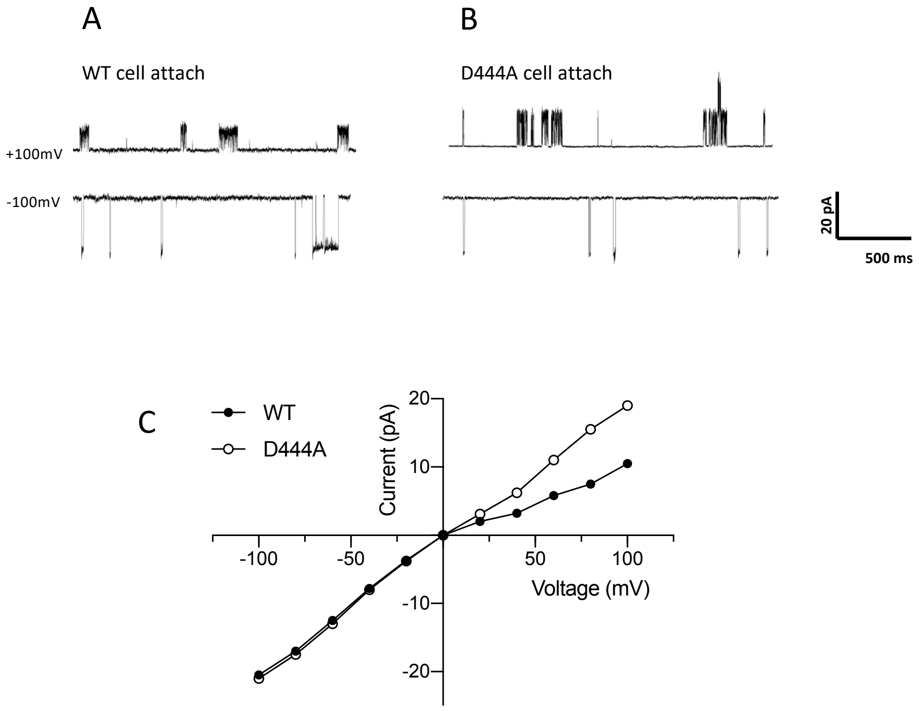

Supplementary Figure 5. D444A mutant, which was mutated at the amino acid residue that directly chelates Mg^{2+}, abolished the cytosolic Mg^{2+}-dependent rectification of the LjCASTOR-2TM channel.
(A & B) Sample traces of LjCASTOR-2TM **(A)** and its D444A mutant **(B)** recorded from cell-attached patches at ±100mV with 150 mM of $[Na^+]$ in the pipette solution.
(C) I-V curves of LjCASTOR-2TM and D444A mutant.

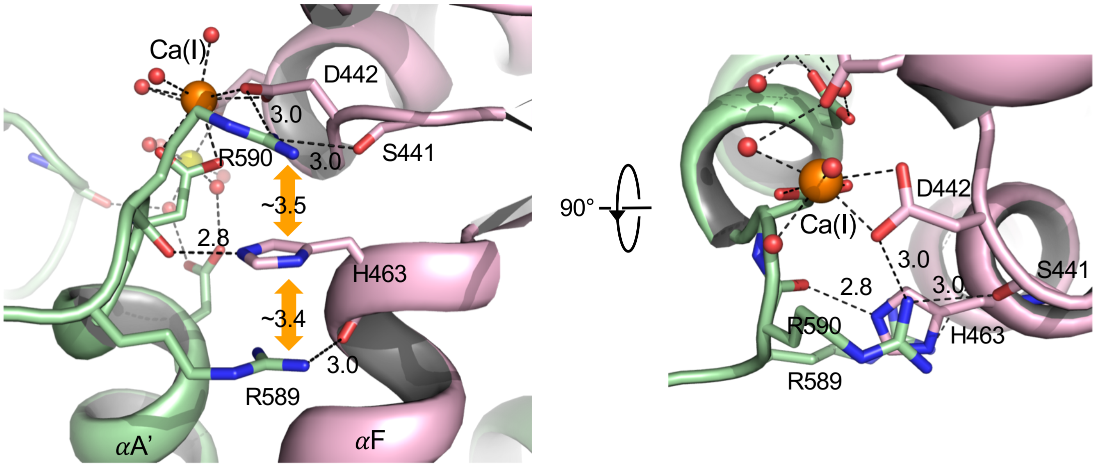

Supplementary Figure 6. π-cation planar stacking interactions near Ca(I) site. The imidazole ring of His 463 (N-lobe of RCK1) inserts into the gap between the sidechains of Arg 589 and 590 (N-lobe of RCK2), and forms π-cation planar stacking interactions as indicated by orange arrows. These three residues also augment the inter-lobe interaction by forming additional hydrogen bonds (black dashed lines) at the interface: R589-H463, R590-S441, and H463-R590. The salt bridge between D442 and R590 further stabilizes the inter-lobe interface by coupling Ca^{2+} coordination at Ca(I) site with π-cation stacking interactions. Numbers indicate distance in Å.

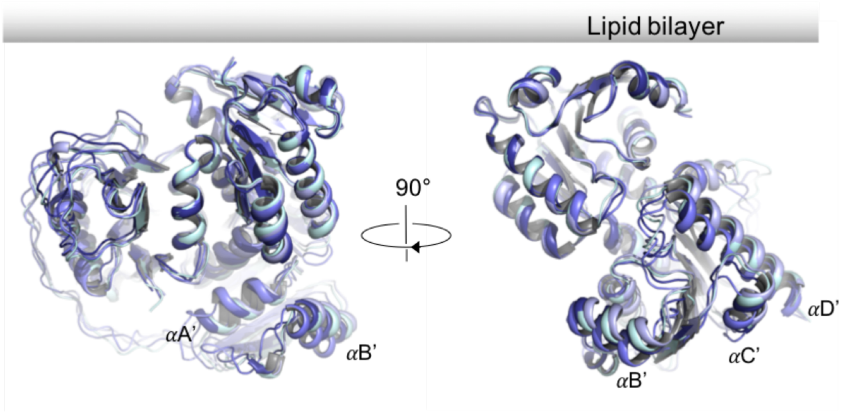

Superimposition of apo subunits A-D

Supplementary Figure 7. Superimposition of the four subunits from the apo LjCASTOR gating ring.

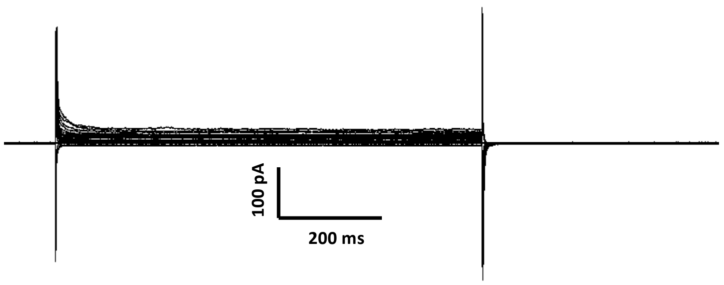

Supplementary Figure 8. No channel activity was observed in the whole cell recordings of the D265N mutant of LjCASTOR-2TM.

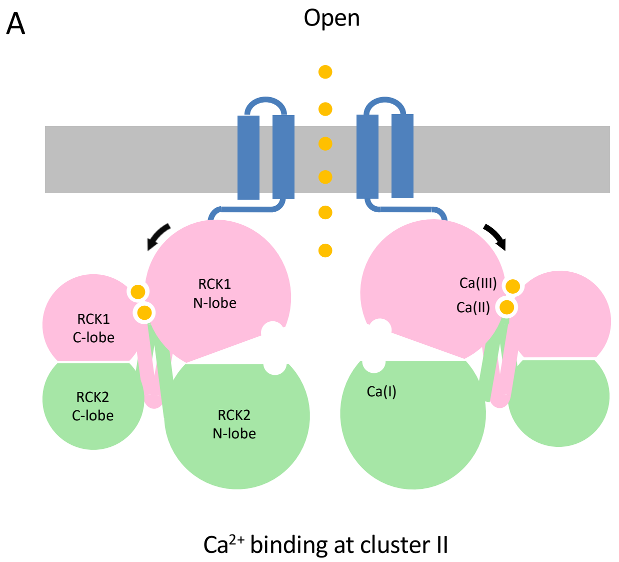

Supplementary Figure 9. Working model of Ca^{2+} regulation of CASTOR.

(A) Ca^{2+} binding at cluster II Ca(II) and Ca(III) sites promotes the expansion of the gating ring and activates the channel.

(B) Ca^{2+} binding at cluster I Ca(I) site stabilizes the inter-lobe interactions between the two N-lobes within each subunit, which would prevent the RCK1 N-lobe from swinging away from the central axis and thereby inhibit channel opening.

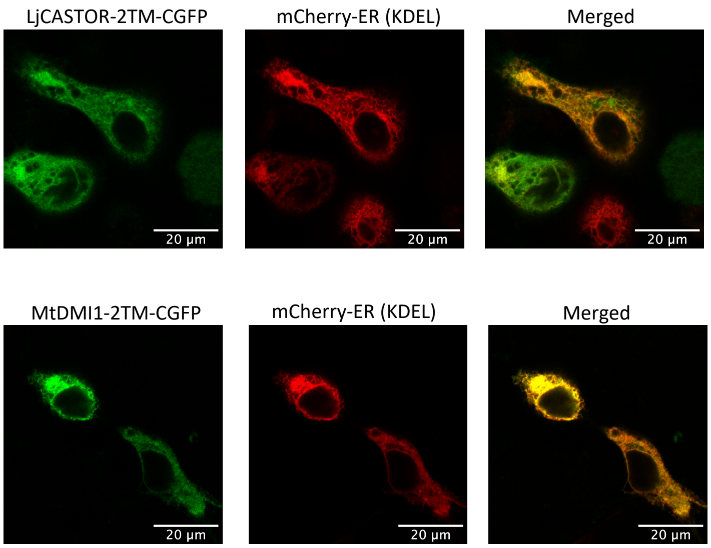

Supplementary Figure 10 Confocal images of HEK 293 cells co-transfected with LjCASTOR-2TM (top) or MtDMI1-2TM (bottom) and ER marker (mCherry-KEDL). The channel proteins were tagged with GFP at C-terminus for localization. A majority of the expressed channel proteins is shown to be localized in ER and nuclear membranes.
