# Supplementary material for: Ca2+-regulated Ca2+ channels with an RCK gating ring control plant symbiotic associations
Source: Nat Commun. 2019 Aug 16;10:3703. doi: 10.1038/s41467-019-11698-5 (PMC6697748; doi:10.1038/s41467-019-11698-5)
Supplement: Supplementary file 3 — Description of Additional Supplementary Files [file 41467_2019_11698_MOESM3_ESM.pdf]

Description of Additional Supplementary Files

File Name: Supplementary Movie 1

Description: The oscillation of the FRET signals from Ca^{2+} sensor (YC3.6) in the HEK 293 cells transfected with wild-type MtDMI1, which reflects the change in the intracellular [Ca^{2+}] in response to the increase in [Ca^{2+}] in the bath solution.
